# Plant miRNA Cross-Kingdom Transfer Targeting Parasitic and Mutualistic Organisms as a Tool to Advance Modern Agriculture

**DOI:** 10.3389/fpls.2020.00930

**Published:** 2020-06-23

**Authors:** Carla Gualtieri, Paola Leonetti, Anca Macovei

**Affiliations:** ^1^Department of Biology and Biotechnology “L. Spallanzani”, University of Pavia, Pavia, Italy; ^2^Institute for Sustainable Plant Protection, National Council of Research, Research Unit of Bari, Bari, Italy

**Keywords:** agriculture, cross-kingdom, microRNAs, mutualism, pathogen, plant

## Abstract

MicroRNAs (miRNAs), defined as small non-coding RNA molecules, are fine regulators of gene expression. In plants, miRNAs are well-known for regulating processes spanning from cell development to biotic and abiotic stress responses. Recently, miRNAs have been investigated for their potential transfer to distantly related organisms where they may exert regulatory functions in a cross-kingdom fashion. Cross-kingdom miRNA transfer has been observed in host-pathogen relations as well as symbiotic or mutualistic relations. All these can have important implications as plant miRNAs can be exploited to inhibit pathogen development or aid mutualistic relations. Similarly, miRNAs from eukaryotic organisms can be transferred to plants, thus suppressing host immunity. This two-way lane could have a significant impact on understanding inter-species relations and, more importantly, could leverage miRNA-based technologies for agricultural practices. Additionally, artificial miRNAs (amiRNAs) produced by engineered plants can be transferred to plant-feeding organisms in order to specifically regulate their cross-kingdom target genes. This minireview provides a brief overview of cross-kingdom plant miRNA transfer, focusing on parasitic and mutualistic relations that can have an impact on agricultural practices and discusses some opportunities related to miRNA-based technologies. Although promising, miRNA cross-kingdom transfer remains a debated argument. Several mechanistic aspects, such as the availability, transfer, and uptake of miRNAs, as well as their potential to alter gene expression in a cross-kingdom manner, remain to be addressed.

## Introduction

Plants have evolved sophisticated mechanisms to adapt to environmental changes and to interact with different organisms. Many of these strategies are based on the activation and repression of large sets of genes, and miRNAs are important regulator molecules in this scenario. They may be induced or repressed to subsequently regulate the expression of target genes through post-transcriptional silencing or translational inhibition of their mRNA targets. MicroRNAs are defined as small, non-coding, single-stranded RNAs acting as regulators of multiple biological and physiological processes. In plants, these small molecules derive from stem-loop precursors that are processed through a Dicer-like (DCL) enzyme and loaded, in association to Argonaute (AGO) proteins, into the RNA-induced silencing complex (RISC) that serve to direct them to their target site where cleavage of mRNAs or inhibition of translation happens (Jones-Rhoades et al., 2006).

Nowadays, miRNAs are starting to be envisioned for their ability to move not only within an organism, but also across kingdoms and influence gene expression in evolutionary distant organisms (LaMonte et al., 2012; Cheng et al., 2013; Shahid et al., 2018; Zhang et al., 2019a). The presence of a methyl group on the ribose of the last nucleotide together with the association with RNA binding proteins and packing into exosomes may contribute to the stability and transfer of plant miRNAs across kingdoms (Valadi et al., 2007; Zhao et al., 2012). The miRNA cross-kingdom transfer may be favored by the conserved features of the RNA silencing machinery among eukaryotes, though taxon-specific variations exist. Such differences are mainly related to the ability of organisms to incorporate RNA molecules, systematically transmit the RNA signals to other tissues and to the magnitude and duration of the RNA silencing response (Winston et al., 2007; Shannon et al., 2008; Wang et al., 2015; Wang et al., 2016). Most examples of miRNA cross-kingdom transfer come from plant-pathogen/parasite interactions (Zhang et al., 2016; Wang et al., 2017a; Zhang et al., 2019a). The cross-kingdom transfer of endogenous plant miRNAs to pathogens or parasites may inhibit their invasive powers while the miRNA transfer from parasitic eukaryotes to plants may suppress the immunity of the host plants. In the case of symbiotic/mutualistic relations, the miRNA transfer from plants may influence essential processes such as growth and development (Zhu et al., 2017).

Understanding the complex network of interactions between plants and eukaryotic organisms and the translation of these information from the bench to the field can pave the way for the development of new technologies. In view of this, miRNA-based strategies exploiting the potential of plant miRNAs to move across kingdoms and silence specific genes in distantly related organisms, are gaining ground. The use of artificial miRNAs (amiRNAs) can be regarded as valuable tools that can complement the already existing technologies to face the global climate changes and associated agricultural challenges (Chen et al., 2013; Mitter et al., 2016).

The current minireview focuses on the latest information related to cross-kingdom miRNA addressing plant-parasite/mutualistic relations. Specific examples of cross-kingdom transferring plant miRNAs and potential gene targeting are provided and their potential implication in improving agricultural practices are discussed. Since this is still a highly debated topic, where both positive and negative results are available with regard to plant miRNA stability, abundance, and especially cross-kingdom targeting ability, several open questions are being proposed relative to methodological and mechanistic issues.

## Plant-Parasite miRNAs Cross-Kingdom Transfer: Alternative Tools to Fight Plant Pests and Diseases

Among plant diseases, agricultural crop infection by fungal pathogens annually cause multimillion dollars losses. While the most used methods to combat fungal-borne diseases are fungicides and chemical sprays, these have negative impacts on human health and surrounding environment (Almeida et al., 2019). The cross-kingdom miRNAs delivery between plants and fungi may represent alternative, environmental-friendly approaches to fight fungal diseases and confer crop protection (Wang et al., 2016). To date, miRNAs have been observed to move in a cross-kingdom manner from plants to fungi and vice versa (**Table 1**). An example of plant miRNA transfer to pathogenic fungi is constituted by miR159 and miR166 from cotton (*Gossypium hirsutum*), shown to confer resistance to *Verticillium dahlia* (Zhang et al., 2016). These miRNAs, found in fungal hyphae isolated from infected cotton tissues, were predicted to hit the virulence-related proteins HiC-15 (isotrichodermin C-15 hydroxylase) and Clp-1 (Ca^2+^-dependent cysteine protease). The targets were validated by transiently expressing miRNA-resistant HiC-15 and Clp-1 in tobacco and *V. dahliae*. Subsequent analysis of *V. dahliae* mutants confirmed that the targeted fungal genes had an important role to play during fungal virulence and that they were specifically targeted by the miRNAs exported from the infected cotton plants to achieve silencing, hence conferring resistance to the fungal pathogen (Zhang et al., 2016). An example of fungal miRNA delivery to host plants is the case of a novel miRNA-like RNA from *Puccinia striiformis* f. sp. *tritici* (*Pst*), the agent causing the wheat stripe rust disease, able to act as a pathogen effector and suppress wheat innate immunity (Wang et al., 2017a). Pst-milR1, identified by high-throughput analysis of *Pst* sRNA library, was predicted to target the β-1,3-glucanase *SM638* (pathogenesis-related 2) gene in wheat. Co-transformation analyses and RACE (rapid amplification of the cDNA ends) validation in tobacco leaves confirmed that *SM638* was targeted by Pst-milR1.

**Table 1:** Examples of cross-kingdom miRNA transfer related to plant parasitic and mutualistic relations.

Donor	Receiver	Relation	miRNA	Target	Function	Reference
***G. hirsutum***	*V. dahlia*	Parasitic	miR159 miR166	*HiC-15* *Clp-1*	Fungal virulence	Zhang et al., 2016
***P. striiformis***	*T. aestivum*	Parasitic	pst-milR1	*SM638*	Innate immunity	Wang et al., 2017a
***A. thaliana***	*P. xylostella*	Parasitic	miR159c novel-7703-5p	*BJHSP1* *PPO2*	Pupae development	Zhang et al., 2019a
***B. campestris***	*A. mellifera*	Mutualistic	miR162a	*TOR*	Caste development	Zhu et al., 2017

When considering insect pests, the cross-kingdom transfer of miRNAs has been investigated for its communication role between plants and plant-feeding insects, such as *Plutella xylostella* (diamondback or cabbage moth) (Zhang et al., 2019a). RNA sequencing analysis has evidenced the presence of 39 plant miRNAs in the moth hemolymph. The plant-derived miR159a, miR166a-3p, and the novel-7703-5p were predicted to influence cellular and metabolic processes in *P. xylostella* through binding and suppressing *BJHSP1, BJHSP2* (basic juvenile hormone-suppressible protein 1 and 2), and *PPO2* (polyphenol oxidase subunit 2) genes. QRT-PCR analyses carried out following treatment with the specific miRNA agomir sequences, demonstrated the downregulation of the predicted targets whereas a luciferase assay proved the binding to their respective targets. Further insect development studies revealed that treatments with agomir-7703-5p resulted in the development of abnormal pupae and decreased adult emergence rates (Zhang et al., 2019a).

Other examples focused on showing the presence of plant-derived miRNAs in insect pests. For instance, Zhang et al. (2012) investigated this aspect in several *Lepidoptera* and *Coleoptera* species subjected to controlled feeding experiments. This study focused on determining the presence of conserved miR168 sequences in insects by means of northern blot and deep sequencing; while northern blot analyses were negative, the deep sequencing data revealed the presence of miR168 in moderate quantities. Hence, the authors discuss the possibility of sample contamination evidencing the existence of some artefacts during sequencing data analysis (Zhang et al., 2012). Deep sequencing was used to reveal plant miRNAs in cereal aphids (*Schizaphis graminum*, *Sipha flava*) causing serious losses in sorghum (*Sorghum bicolor*) and switchgrass (*Panicum virgatum*) crops (Wang et al., 2017b). Thirteen sorghum miRNAs and three barley miRNAs were detected and predicted to target aphid genes playing important roles in detoxification, starch and sucrose metabolism.

MiRNA cross-kingdom transfer probably occurs also during the interplay between plants and parasitic nematodes (phytonematodes) (Jaubert-Possamai et al., 2019). Plant-parasitic nematodes are responsible for considerable crop losses worldwide. Understanding how plants respond to these organisms is necessary to bridge the gap between agricultural production and the growing food demand. Most of the scientific literature on gene silencing mechanisms comes from nematodes, specifically from *Caenorabditis elegans*. However, these studies mostly focus on the ability to uptake double strand RNAs (dsRNAs) from the environment (Huang et al., 2006; Tian et al., 2019) rather than on the cross-kingdom transfer of plant miRNAs. Many studies have investigated the involvement of plant miRNAs and their corresponded gene targets in response to phytonematodes infection (Hewezi et al., 2008; Li et al., 2012; Lei et al., 2019; Pan et al., 2019). Transcriptomic analyses evidenced extensive reprogramming of gene expression at the nematode feeding sites, modulated by plant miRNAs; also, some conserved miRNAs were shown to have analogous roles in feeding site formation in different plant species (Jaubert-Possamai et al., 2019).

The cited examples depict a promising research area. Understanding the complex interactions between host plants and parasitic organisms would pave the way for the development of new technologies for a more sustainable control of plant pests and diseases.

## MiRNAs Cross-Kingdom Transfer in Plant Mutualistic Interactions

Several studies on mutualistic relations have regarded many miRNAs target processes related to hormone-responsive pathways and innate immune function (Formey et al., 2014; Wu et al., 2016). The majority of these processes correspond to turning off defense pathways that would otherwise block fungal or bacterial proliferation within plant tissues (Plett and Martin, 2018). In a recent study, Silvestri et al. (2019) have looked into the symbiosis between the arbuscular mycorrhiza (AM) *Rhizophagus irregularis* and the model legume *Medicago truncatula*, showing the presence of fungal microRNA-like sequences potentially able to target plant transcripts. The *in silico* analysis, verified through a degradome analysis, predicted more than 200 plant genes as putative targets of specific fungal sRNAs and miRNAs, many of which had specific roles in AM symbiosis. For instance, three miRNA-like sequences (*Rir*-miRNA-like 341, 342, and 828) shown to be up-regulated in the intraradical phase were suggested to be responsible for the regulation of AMF genes required to manipulate fungal or host plant gene expression. The predicted target genes encode for a DHHC-type zinc finger protein (AES89412), integral membrane family protein (AES91391), and carboxy-terminal region remorin (AES81367).

In recent years, evidence that plant miRNAs target genes in a trans-kingdom fashion in pollinator insects is steadily accumulating. Currently available studies report pre-eminently on dietary intake of plant miRNAs by honey bees (*Apis mellifera*) (Ashby et al., 2016; Gismondi et al., 2017; Zhu et al., 2017). The plant-pollinator relationship is partly mutualistic considering the nutrients intake in exchange for the pollination service that enables plant reproduction. The presence of plant miRNAs in honey was reported by Gismondi and collegues (2017) who detected and quantified several miRNAs belonging to conserved families (miR482b, miR156a, miR396c, miR171a, miR858, miR162a, miR159c, miR395a, miR2118a) in different types of honey. The authors found that the most enriched in plant-miRNAs was the honey obtained from sweet chestnut (*Castanea sativa*) flowers. In bees, the dietary intake of pollen-derived miR162a was proven to regulate caste development at larval stage (Zhu et al., 2017). It was shown that miR162a targets *TOR* (target of rapamycin) mRNA downregulating its expression at the post-transcriptional level. Interestingly, this mechanism was found to be conserved in *Drosophila melanogaster* (common fruit fly), a non-social type of insect (Zhu et al., 2017). Nonetheless, contrasting results are also reported. Although Masood et al. (2016) observed accumulation of plant miRNAs after pollen ingestion in adult bees, they did not find any evidence of biologically relevant roles of these plant miRNAs in bees. Likewise, expression analysis of pollen-derived miRNAs ingested by bees, revealed the absence of substantial uptake and systemic delivery of miR156a, highly expressed in bee-bread and honey (Snow et al., 2013). In a different system, silkworm (*Bombyx mori*) and mulberry (*Morus* spp.) was used as model to study the proposed miRNA-mediated crosstalk between plants and insects (Jia et al., 2015). Sanger sequencing and digital PCR demonstrated the presence of mulberry-derived miRNAs in silkworm tissues while the administration of synthetic miR166b did not influence silkworm physiological progress.

## Cross-Kingdom Transfer of amiRNAs for Agricultural Purposes

The knowledge acquired on endogenous miRNAs as regulators of gene functions within and among organisms led researchers to develop increasingly sophisticated agricultural technologies based on miRNAs. Among these, the amiRNA (artificial miRNA) strategy was developed to produce specific miRNAs that can effectively silence designated genes (Zhang et al., 2018). One of the main characteristics of amiRNAs is the conserved secondary foldback structure that has to be similar to that of a typical pre-miRNA. In this case, the original structure of the miRNA-5p:miRNA-3p sequence will be replaced by an engineered miRNA targeting a designated mRNA, and the most preferred structures are those existing in conserved miRNA families. In this way, amiRNAs can be engineered to target any mRNA with higher specificity compared to other strategies like dsRNA overexpression or siRNA accumulation. Since pre-amiRNA processing results in a single amiRNA targeting a designated sequence, this eliminates the off-target effects and the production of secondary siRNAs is quite limited (Manavella et al., 2012). A highly relevant attribute for agricultural purposes is the fact that amiRNAs are stable and inheritable. Moreover, the amiRNA-mediated silencing is believed to pose less problems regarding bio-safety and environmental security with respect to other strategies (Liu and Chen, 2010; Toppino et al., 2011), due to the small size of the inserts and reduced probabilities for horizontal transfer. Aside the study of gene functionality (Schwab et al., 2006; Warthmann et al., 2008), amiRNA technology has been applied to knock out genes from insect pests, nematodes, viruses, and other phytopathogens (Niu et al., 2006; Fahim et al., 2012; Guo et al., 2014; Kis et al., 2016; Wagaba et al., 2016).

Several pre-miRNAs have been used as backbones to synthesize artificial miRNAs in engineered plants with the aim to control agricultural pests (**Table 2**). This strategy is based on the possibility of miRNAs to be transferred through diet across kingdoms and the ability of these small molecules to exercise their biological activity in recipient organisms. Indeed, the miRNAs in the transgenic plant may be taken up by plant feeding organisms and then suppress selected genes such as those related to metabolism, development but also to pathogenesis/parasitism by exploiting the endogenous silencing machinery of the plant feeding organism. Essential genes either involved in pathogen metabolism, or causing resistance to plant toxins, or encoding effectors involved in pathogenicity, have been considered as potential targets. For instance, enhanced resistance to the aphid *Myzus persicae* was reported in transgenic plants expressing amiRNAs targeting the *MpAChE2* (aphid acetylcholinesterase 2) gene (Guo et al., 2014). The *AChE* gene encodes for hydrolase enzyme that hydrolyses the neurotransmitter acetylcholine and plays vital roles in insect growth and development (Kumar et al., 2009). In a recent investigation, amiRNA-based technology targeting AChE was also applied by Saini and co-workers (2018) to defeat *Helicoverpa armigera*. They demonstrated that the silencing of *HaAce1* gene by host-delivered amiRNAs disrupted growth and development in the polyphagous insect. Another example relates to the use of amiR-24 targeting the 3′-UTR of the *chitinase* gene. Transgenic tobacco plants producing amiR‐24 were fed *H. armigera* caterpillars, resulting in delayed molting and enhanced lethality (Agrawal et al., 2015). In a different study, amiR15 was used to design transgenic rice plants resistant to the striped stem borer, *Chilo suppressalis* (Jiang et al., 2016). The amiR15, design staring from the insect specific miRNA, *Csu*‐miR‐15, targets the *CsSpo* (Cytochrome P450 307a1) and *CsEcR* (Ecdysone receptor) genes involved in the ecdysone signaling pathway. Feeding trials carried out using the transgenic miR‐15 rice resulted into increased mortality and developmental defects in the targeted insect pest. The effect of amiRNAs was studied also on the *Avr3a* gene, the target transcript of *Phytophtora infestans*. AmiRNAs targeting different regions of the *Avr3a* gene imparted moderate type of late blight resistance into two transformed Indian potato cultivars (Thakur et al., 2015).

**Table 2:** Examples of cross-kingdom transfer of artificial microRNAs (amiRNAs) from transgenic plants to their respective pathogens/parasites.

Modified plant	Pathogen/parasite	Target	Function	Reference
***N. tabacum***	*M. persicae*	*MpAChE2*	Synaptic transmission	Guo et al., 2014
***A. thaliana***	*H. armigera*	*HaAce1*	Synaptic transmission	Saini et al., 2018
***N. tabacum***	*H. armigera*	*Chitinase*	Chitin synthesis	Agrawal et al., 2015
***O. sativa***	*C. suppressalis*	*CsSpo CsEcR*	Embryonic development	Jiang et al., 2016
***S. tuberosum***	*P. infestans*	*Avr3a*	Fungal virulence	Thakur et al., 2015

AmiRNA delivery may be considered as a species-specific pesticide and as a potential and powerful alternative to the chemical strategies used so far. This miRNA-based technology may be considered as an alternative method for intragenic crop engineering causing less public concern. For beneficial insects, such as honey bees, amiRNA-based technology may be used to counteract virus infections by feeding them in large field treatment with amiRNAs able to reduce the expression of viral genes. Apart from transgenic plants permanently expressing amiRNAs, amiRNAs sprayed onto leaves in conjunction with miRNAs enriched soil can minimize pest damages (Cagliari et al., 2019).

## Conclusions and Future Perspectives

Plant pathogens place a global burden on major crops being responsible for reduced crop yields with great repercussions on food production and food security (Savary et al., 2019). On the other hand, promoting the investigation of mutualistic relations has the potential to better assist sustainable agricultural practices (Duhamel and Vandenkoornhuyse, 2013).

As shown in the presented examples, understanding the miRNA cross-kingdom transfer and mode of action could contribute to decrease the pathogenicity of fungi and pests, hence promoting better plant productivity. In the case of insects (pests or pollinators), administration of plant miRNAs (through genetic engineering, nanoparticles, or spraying) may actively contribute to population control, reducing the prevalence of pests while enhancing the preponderance of pollinators. In this context, researches could be envisioned to grasp on how plant miRNA trans-kingdom regulation could be used to avoid the extinction of bees, as exemplified in the studies demonstrating their involvement in cast development (Zhu et al., 2017). But, to progress this debated field, many questions still need to be addressed and many additional steps must be taken to elucidate plant miRNAs uptake and potential cross-kingdom gene targeting. In this highly-technological era, the rapid progress of bioinformatics studies and tools to predict cross-kingdom miRNA targets (Mal et al., 2018; Bellato et al., 2019) sets the stage to advance new hypothesis to be subsequently experimentally tested. Nonetheless, many of the existing questions demand solid proofs from wet lab analyses. From the point of view of experimental design, questions related to the most appropriate techniques (deep sequencing, digital PCR, qRT-PCR) and references (samples and/or genes) to be used for cross-kingdom miRNA studies still need to be addressed and uniformized accordingly (Chan and Snow, 2016; Zhang et al., 2019b). Once these issues are settled, we can then proceed to investigate other challenges; for instance, why some plant miRNAs seem to be more stable and abundant than others? Are the levels of host plant miRNAs found in pathogen species high enough to exert a physiological impact? How do plant miRNAs reach their targets in the receiving organism? Considering the miRNAs mode of action (targeting mRNAs based on sequence complementarity), their impact on the receiving organism can variate depending on the targeted genes; hence, studies covering both favorable and unfavorable effects need to be encouraged to promote best-informed scientific solutions.

## Author Contributions

AM conceptualized the minireview. CG, PL, and AM wrote the manuscript.

## Funding

AM and CG acknowledge the funding received from FFABR-ANVUR (Funding for Basic Activities Related to Research-Italian National Agency for the Evaluation of Universities and Research Institutes) and the Italian Ministry of Education, University and Research (MIUR): *Dipartimenti di Eccellenza* Program (2018–2022) - Dept. of Biology and Biotechnology “L. Spallanzani”, University of Pavia. The work of PL is framed into the Project “NUTR-AGE” (Progetto FOE 2019, Nutrizione, Alimentazione & Invecchiamento Attivo) funded by the MIUR at the DiSBA-CNR.

## Conflict of Interest

The authors declare that the research was conducted in the absence of any commercial or financial relationships that could be construed as a potential conflict of interest.
